# Use of Virtual Reality and Augmented Reality Technologies to Support Resilience and Skill-Building in Caregivers of Persons With Dementia: A Scoping Review

**DOI:** 10.7759/cureus.64082

**Published:** 2024-07-08

**Authors:** Kristina M Kokorelias, Mary Chiu, Sayani Paul, Lynn Zhu, Nusrat Choudhury, Cole G Craven, Adam Dubrowski, Tyler Redublo, Bill Kapralos, Michael S.D. Smith, Adriana Shnall, Joel Sadavoy, Amer Burhan

**Affiliations:** 1 Section of Geriatrics, Sinai Health and University Health Network, Toronto, CAN; 2 Rehabilitation Sciences Institute, Temerty Faculty of Medicine, University of Toronto, Toronto, CAN; 3 Research & Academics, Ontario Shores Centre for Mental Health Sciences, Whitby, CAN; 4 Faculty of Health Sciences, Ontario Tech University, Oshawa, CAN; 5 Medical Devices, National Research Council Canada, Boucherville, CAN; 6 Computer Science, Ontario Tech University, Oshawa, CAN; 7 maxSIMhealth Group, Ontario Tech University, Oshawa, CAN; 8 Translational Research Program, Temerty Faculty of Medicine, University of Toronto, Toronto, CAN; 9 Software Informatics Research Centre, Ontario Tech University, Oshawa, CAN; 10 Medical Devices, National Research Council Canada, Winnipeg, CAN; 11 The Koschitzky Centre for Innovations in Caregiving, Baycrest Centre, Toronto, CAN; 12 Factor Inwentash Faculty of Social Work, University of Toronto, Toronto, CAN; 13 Department of Geriatric Psychiatry, Mount Sinai Hospital, Toronto, CAN; 14 Department of Psychiatry, Temerty Faculty of Medicine, Unviersity of Toronto, Toronto, CAN; 15 Applied Mental Health, Ontario Shores Centre for Mental Health Sciences, Toronto, CAN; 16 Department of Psychiatry, Temerty Faculty of Medicine, University of Toronto, Toronto, CAN

**Keywords:** caregiver training innovations, dementia care, family caregiver, immersive technology, intervention, virtual reality simulation

## Abstract

Dementia presents a growing public health challenge with most affected individuals living at home, placing significant responsibility on their caregivers. Various interventions, from traditional support groups and education programs to emerging technologies, and more specifically virtual reality (VR) and augmented reality (AR), aim to enhance caregiver skills. While VR/AR shows promise in educating and fostering empathy among caregivers and healthcare professionals, its overall effectiveness and practicality in older adults and dementia care warrant further exploration.

This review aimed to summarize currently available VR and AR interventions tailored for family caregivers of persons living with dementia (PLWD) in home or clinical settings, including their level of effectiveness, and to compile a summary of features that contributed to technology acceptance in family caregivers of PLWD.

We conducted a systematic search in OVID PsychInfo, CINAHL, Google Scholar, and ERIC, as well as CADTH’s Grey Matters, OpenGrey, National Technical Information Service, OAIster, and Health Quality Ontario, to comprehensively summarize the existing evidence underscoring the role of VR and AR in supporting education, resilience-building, and skills training for family caregivers of PLWD. The search terms were built with the assistance of a research librarian and involved synonyms for VR, AR, and dementia. Two screeners conducted a rigorous screening and data extraction to analyze and summarize findings. Studies were included if they focused on family caregivers engaging in interventions utilizing a three-dimensional VR environment and/or Metaverse for group learning in psychotherapeutic modalities such as psychoeducation, therapy, communication, and skill-building. The primary outcome of the studies was assessing measures of well-being (e.g., quality of life, communication, interaction, personhood) and learning outcomes for caregivers, while the secondary outcomes focused on identifying barriers and facilitators influencing the acceptability of VR/AR among dementia caregivers. Content analysis and descriptive statistics were used to summarize key trends in technology and evidence effectiveness and acceptability.

Of the 1,641 articles found, 112 were included, with six articles meeting inclusion for analysis. Studies differed in duration and frequency of data collection, with interventions varying from single events to months-long programs, often employing home-based approaches using VR or online platforms. No study used AR. Usability issues and unclear benefits of use were identified as factors that hinder technology acceptance for dementia caregivers. However, technologies demonstrated engaging user experiences, fostering skill-building, confidence, and competence among caregivers. Positive psychological effects were also observed, facilitated by immersive VR and AR interventions, resulting in improved caregiver empathy and reduced stress, depression, and loneliness.

VR and AR interventions for family caregivers of PLWD show the potential to enhance empathy and skills and reduce stress. Challenges such as technological limitations and user inexperience issues persist. Home-based VR training aligns with caregiver comfort but lacks focus on financial aspects and cultural competencies. Co-design approaches offer solutions by addressing user concerns and promoting end-user engagement or empowerment.

## Introduction and background

Dementia is a major public health challenge that continues to grow as the number of persons living with dementia (PLWD) increases with the aging population [1]. About 61% of PLWD live at home in contrast to long-term care institutions, placing a great deal of responsibility on their family caregivers [2]. Caring for a family member with dementia leads to disproportionate vulnerability to physical, mental, and social adverse health consequences among family caregivers [3]. Proper family caregiver skills training may positively impact a variety of well-being metrics, such as resilience, mental health, and the ability to manage caring for a PLWD [4]. Dementia also disproportionately strains the emotional life and productivity of employed family caregivers [5].

Existing clinical and traditional interventions for family caregivers have played a pivotal role in supporting those who provide care to an older adult with dementia. These interventions encompass a range of strategies, from support groups and counseling to educational programs and respite care [6]. Additionally, respite care offers caregivers much-needed breaks, which may mitigate risks of physical and emotional exhaustion [7]. Interventions are most effective when they are tailored to the evolving needs of family caregivers [8]. For example, traditional interventions such as facilitated support groups create a sense of community and provide caregivers with an opportunity to share their experiences and gain valuable insights from others facing similar situations [9]. Psychoeducational programs equip caregivers with the knowledge and skills needed to provide tangible hands-on care [10].

Several technological interventions, including virtual reality (VR) and augmented reality (AR) simulations, have been developed in recent years to allow healthcare professionals, students, and both professional and non-professional caregivers (i.e., family caregivers) the opportunity to deeply engage with the lived experiences of PLWD [11]. While the use of VR simulation in dementia care is a relatively recent innovation with limited information regarding its efficacy, early indicators suggest that it is an effective tool for both educating and engendering empathy toward PLWD among healthcare professionals and family caregivers [12]. A primary factor that promotes empathy among those who care for PLWD is the first-person perspective that an embodied experience affords [13]. This is significant because empathy is central to the principles of person-centered care, which is the gold standard in dementia care based on the individual needs of PLWD that are identified through interpersonal relationships [14].

VR and AR interventions have been shown to be efficacious in terms of learning outcomes for caregivers and can promote behavioral change in PLWD [15]. These technologies allow caregivers to safely experience a wide range of real-world situations, adjust for communication barriers, maintain user anonymity, and foster therapeutic relationships [15]. In the context of this review, we define VR and AR interventions as those that involve immersing users in a computer-generated, interactive, and often simulated environment. AR interventions overlay digital information and virtual objects onto the real world, enhancing the user’s perception of their physical surroundings [16]. However, VR immerses users in a computer-generated environment, typically isolating them from the physical world, providing a fully synthetic experience [17]. In contrast, AR overlays digital content onto the real-world environment, enhancing the user’s perception of their physical surroundings by integrating computer-generated elements with their immediate surroundings [18].

Recently, scoping reviews have begun to explore the use of VR and AR in the context of dementia education. A 2023 review focused on AR in dementia care concluded that there has been limited research to establish the practicality, safety, and acceptability of AR use in older adults, especially those with dementia [18]. Nevertheless, due to its potential for being a safer, user-friendly, and easily deployable technology, it could prove beneficial as a supplementary tool for various applications in the field of dementia care [18]. Jones et al. [17] undertook a review of 19 qualitative, quantitative, and mixed-method studies published between January 1, 2000, and May 31, 2021 [17]. The studies were based on five types of VR and AR simulations and target participants included students, healthcare professionals, instructors, and formal and family caregivers [17]. While the authors noted the studies showed mixed results, they reported positive effects for the participants. The immersive VR-based interventions in the review demonstrated their impact on empathy in several ways. Users reported increased knowledge of dementia, new approaches to care for PLWD, a shift toward more positive attitudes toward PLWD, greater confidence and competence in providing care, and heightened empathy and sensitivity toward both PLWD and their specific care requirements [17]. Users also learned new approaches to care for PLWD [17]. An earlier 2020 review of six studies undertaken between 2012 and 2017 offered an overview of VR simulations in dementia caregiving [19]. The authors’ assessment of the simulations’ impact on caregivers was based on eight criteria, namely, empathy, perceived pressure from care, perceived competence, competence, quality of the relationship between the caregiver and PLWD at home, loneliness, depressive mood, caregiver burden, and stress [19]. Measurement of the learning process was based on perceived confidence, competence, and compassion [19]. While the articles included in the review reported that improvements in caregiver empathy were achieved, the authors also noted an absence of controlled design, suggesting that there is much room for improvement when evaluating the effectiveness of VR simulations in dementia care [19]. Garcia-Betances et al. [20] undertook a review of various VR products, categorizing them by purpose, i.e., diagnosis, cognitive training, and caregiver education [20]. The review suggested that, to maximize the potential of VR products, developers should incorporate the latest display, interaction, and feedback technologies and that where possible they should be designed to allow for use in both long-term care home and private household settings [20]. Recent advancements in VR technology have responded to such barriers with improved affordability, portability, and tracking features [20], facilitating autonomous use.

While there have been reviews that touched on the use of VR and AR in dementia education and caregiver support, the current review narrows its scope to address the unique needs of family caregivers caring for PLWD in either home or facility settings. This narrower focus allows this review to delve deeper into the specific challenges and benefits of VR and AR interventions in this context, providing a more comprehensive understanding of the potential applications and limitations of these technologies for family caregivers. By emphasizing the family caregiver’s perspective and their need for psychoeducation and skills training, the current review aims to offer a more nuanced analysis of the existing literature, shedding light on the gaps and opportunities in the implementation of VR and AR to support caregivers. Thus, the objectives of this review were to (1) identify VR and AR-enabled solutions, programs, or interventions that have been tested with family caregivers of PLWD; (2) provide an overview of the benefits described in the literature; and (3) summarize the barriers and facilitators related to technology acceptance in family caregivers of PLWD. In the context of this review, effectiveness refers to the extent to which an intervention produces desired outcomes in real-world settings, focusing on its impact under usual or everyday conditions [21]. On the other hand, implementation differences pertain to the strategies and factors influencing the successful integration of an evidence-based intervention into routine practice [21,22]. Technology acceptability as an implementation outcome refers to the degree to which individuals or organizations are willing to embrace and use a particular technology in a given context.

The current review aimed to summarize the use of VR and AR to support family caregivers in caring for PLWD who live in the community or a facility. Specifically, this review aims to build upon existing reviews in the field of VR and AR interventions for family caregivers of PLWD, with a specific focus on the effectiveness of these inventions, and the barriers and facilitators to acceptability and implementation as a form of caregiver psychoeducation and skills training.

## Review

Methodology

We examined the literature on the use of VR and AR to support education, resilience-building, and skills training in family caregivers caring for PLWD. We selected a scoping review methodology as this approach plays a crucial role in summarizing broad and diverse subjects, particularly those related to implementation considerations [23]. Our scoping review followed the methodological steps by Arksey and O’Malley [23] and refinements by Levac et al. [24]. Thus, this review included the following five steps: (1) identify the research question; (2) identify relevant studies; (3) select studies; (4) chart, collate, and summarize the data; and (5) report results [23]. A protocol was registered with the Open Science Forum (osf.io/7fyt) in February 2023. No deviations to the protocol were made, except that we extended the search to October 2023 from January 2023. We report our review in adherence to guidelines for scoping reviews and Preferred Reporting Items for Systematic Reviews and Meta-Analyses extension for Scoping Reviews (the PRISMA-ScR) [25].

Identifying the Research Question

Our research questions were (1) what VR and AR-enabled solutions, programs, or interventions have been tested in family caregivers of PLWD? (2) Did these interventions demonstrate effective outcomes?; (2a) Yf so, what were they? (3) What are the barriers and facilitators for VR and AR-enabled interventions related to technology acceptance in family caregivers of PLWD?

Identifying Relevant Studies

We sought relevant peer-reviewed articles and grey literature in the English language published between January 2010 and October 2023 to capture the most up-to-date technologies. Databases were searched using text words and, wherever possible, subject headings and validated searches, developed by a clinical research librarian. In consultation with the information specialist, a search was first developed in Medline by the research team while planning the full-scale review. Once finalized, this was adapted for use in all other databases. In addition to Medline, we searched OVID PsychInfo, CINAHL, Google Scholar, and ERIC. In addition to searching the noted databases, we conducted searches for grey literature in CADTH’s (Canadian Agency for Drugs and Technologies in Health) Grey Matters, OpenGrey, National Technical Information Service, OAIster, and Health Quality Ontario (HQO), including Ontario Health Technology Assessment Series. The Information Specialist conducted all searches and imported all articles to an EndNote library created for the study. Articles were then de-duplicated using the Bramer method [26]. The final list of unique studies was then imported into Covidence, a review management online platform, to facilitate screening [27]. To ensure a comprehensive search was conducted, we facilitated a hand search of the reference list of the included articles for relevant evidence sources and conducted forward-searching [28].

Study Selection

Study selection occurred in two stages, namely, a title and abstract review, and a full-text review. Both stages were conducted in duplicate by the first author and a research assistant who reviewed each article to determine eligibility based on our inclusion criteria (Table 1). The authors had good inter-rater reliability [29] (i.e., kappa statistic >0.62) [30]. Given that the nature of this review allows for a degree of variability in interpretation, we mitigated subjectivity through safeguards such as tie-breaker mechanisms (i.e., having a third voter) and group discussions. All discrepancies were resolved by a third reviewer (MC) keeping in mind the inclusion and exclusion criteria and team discussion. Following the Levac et al. [24] recommendation, the final inclusion and exclusion criteria were developed based on an iterative process of group discussion during regular team meetings throughout various stages of the process. Inclusion and Exclusion criteria are detailed in Table 1.

**Table 1:** Inclusion and exclusion criteria.

Study element	Inclusion criteria	Exclusion criteria
Participants	Caregiver, care partners, carers, family members, unpaid caregivers	If the focus is on the person living with dementia
Study design	Any empirical study design	Reviews, commentaries, editorials
Technological features	Use of a 3D virtual reality environment; Metaverse (group learning); multi-user; augmented reality	Virtual reality was not used in virtual reality-based interventions, for example, 2D tablet/conventional computer screen display
Treatment modality	Psychotherapy, psychoeducation, therapy, communication, problem-solving, skill-building, applied learning	
Outcome	Primary outcome: quantitative or qualitative outcomes of well-being (e.g., quality of life, communication, interaction, personhood) or learning-related outcomes for carers as a primary, secondary, or tertiary study objective. Secondary outcome: Barriers and facilitators affecting virtual reality/augmented reality acceptability by dementia carers	Interventions targeting only cognitive or memory improvement, physical rehabilitation, reviews/technology appraisals in persons living with dementia

Data Charting

A data extraction form was developed for the charting of relevant data from the included articles (see Table 2). Two reviewers extracted the data in duplicate from the included studies independently using a pilot-tested form on Covidence (KMK and MJ or KMK and TR). This was reviewed for consistency by a third reviewer (MC).

**Table 2:** Data extraction template.

Extracted data	Details
Publication information	Authors, title of the article/journal, year of publication, countries where the study was conducted
Outcomes of well-being and learning	Reported effectiveness of the intervention on outcomes informed by Hirt and Beer [19]: caregiver competence, resilience, empathy, caregiver burden, skill-building, communication, problem-solving; social (personhood, communication/interaction/relationship), emotional (pain, quality of life)
Study sample	Caregiver typology (spouse, adult child, employed caregivers), age, living arrangement (remote, live together), criteria for the diagnosis of dementia in persons living with dementia (PLWD), and any comorbidities listed as inclusion or exclusion criteria
Intervention administration	Setting, administering person, frequency (sessions per week), duration (of each session), and length of the full intervention (days, weeks, months)
Experimental methods	Reported sample size, study design, data collection methods (observation, survey, interview, etc.), validated instruments used, types of data collected (subjective/objective), caregiver/PLWD feedback, comparison therapy/arm
Virtual reality technical properties	Device(s) used, manufacturer or brand, product name, degrees of freedom, senses stimulated, content, virtual environment (passive vs active), feelings of presence, dementia-related adjustments
Barriers to technology acceptability	Barriers and facilitators affecting virtual reality/augmented reality acceptability by dementia caregivers

Data Summary and Synthesis of Results

As the purpose of scoping reviews is to provide an overview of concepts found in the existing research on a topic [23], we report our results in the sections below using the charted findings as tables in our synthesis. Descriptive statistics were used to understand the characteristics of the articles. For the data on the purpose of the studies, concepts and topics addressed, and results, we used a qualitative directed content analysis [31] to provide a content summary of the included data. Through the data analysis process, we held regular team meetings informed by the Arksey and O’Malley framework [23] to discuss identified evidence gaps, future research opportunities, and implications for policy or practice. Nuances between the studies were noted throughout these team meetings.

Results

**Figure 1:**
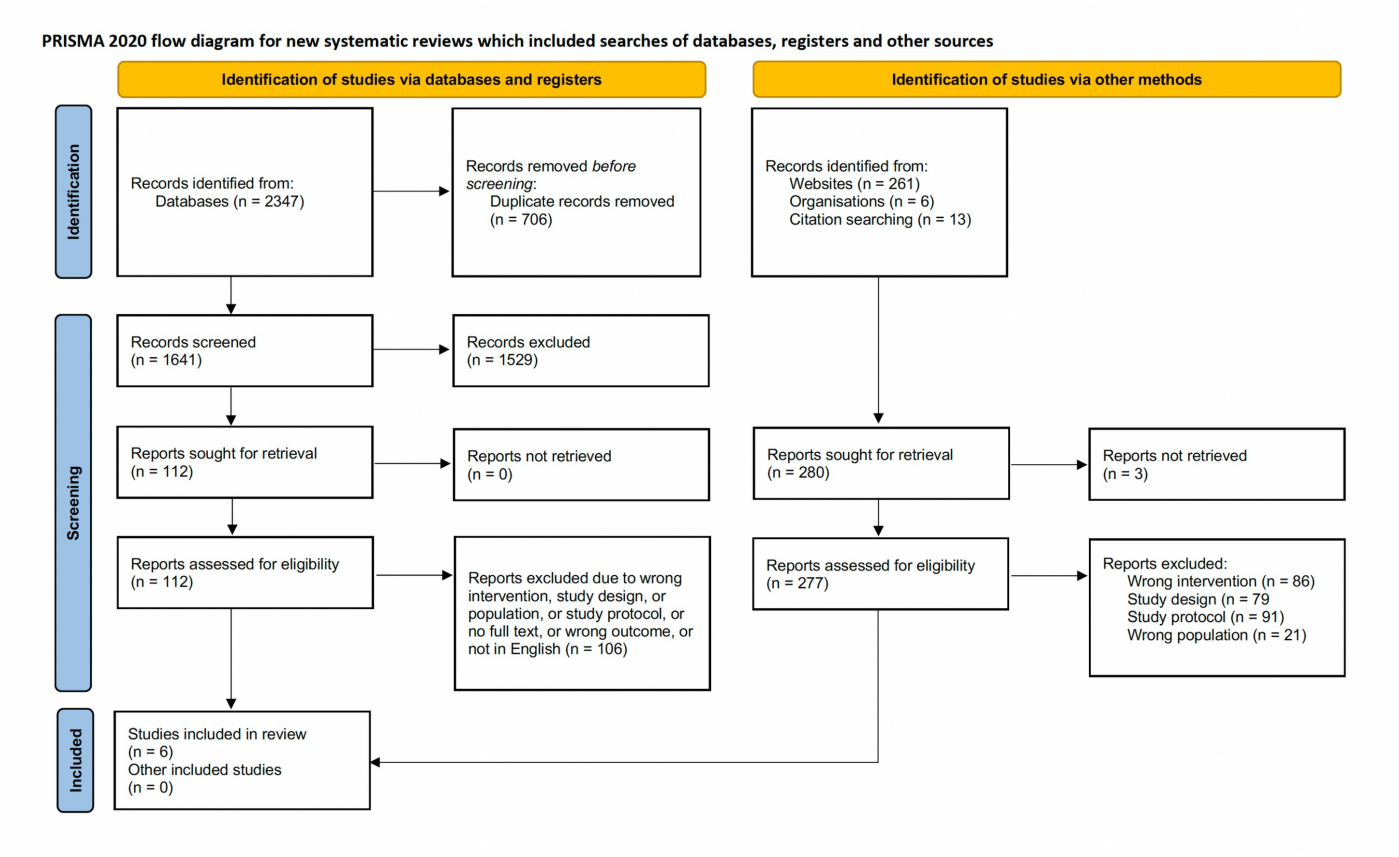
Preferred Reporting Items for Systematic Reviews and Meta-Analyses flow diagram

The database search identified 1,641 unique peer-reviewed articles that were screened for eligibility. Following the title/abstract review, the full text of 112 articles was reviewed. In the end, four of the articles that met the inclusion criteria came from the database searches and two sources from the hand searches (see Figure 1: PRISMA flow diagram). Eligible grey literature was not found. These articles were published from various locations, including the United States, the United K, Ireland, Australia, and the Netherlands. No study used AR. Consequently, the results and discussion of our study will primarily focus on VR interventions. However, it is crucial to underscore that the absence of research concerning AR represents a significant gap in the literature. The study designs within the articles ranged from pilot interventions to randomized controlled trials and qualitative studies. Several barriers to technology acceptance were identified. These included users with physical limitations such as a limited range of motion, challenges using technology due to a lack of familiarity, and lack of user-friendliness and intuitiveness. Despite the barriers, VR technologies offered various benefits. Users generally showed a high degree of engagement with the technology, and caregivers expressed positive psychological effects and reduced stress, depression, and loneliness. Improved empathy, confidence, and competence were also observed. The subsequent sections will present an overview of the study characteristics, an examination of the intervention characteristics from all the reviewed studies, and an analysis of the main themes identified in the findings.

Intervention Characteristics

The level of detail and reporting varied across interventions, with some lacking specific information about the duration, intervention, and specificity as to whether it was VR or AR. The duration of the VR and AR experience ranged from a single event for a few hours to over three months. Both Slater et al. [32] and Flynn et al. [33] employed interventions of 2.5 and 2 hours, respectively. Currie-Harrington et al. [34] conducted 10 face-to-face interviews with no exact duration of the study itself given. The frequency of data collection across the studies also varied significantly, with most studies collecting data once, regardless of the study’s overall duration. Table 3 outlines the intervention characteristics.

**Table 3:** Intervention characteristics.

Authors, year, country, study design	Intervention name	Sample size and intervention description	Study objective	Intervention objective	Intervention duration	Intervention frequency	Primary mode of delivery	Technical properties	Intervention outcomes on caregivers
Flynn et al. [33]. Country: Ireland. Design: technology probe	Introduction and onboarding to virtual reality (VR)	Nine family caregivers and persons living with dementia (PLWD) dyads. PLWD and their caregivers were introduced to VR to inform the future design of a VR application	To evaluate the use of a technology probe, VR FOUNDations, which enables PLWD and their caregivers to familiarize themselves with the functionalities of VR, in this case, to inspire the design of a bespoke VR application	To enrich the social connectedness of older adults living with dementia and their caregivers	Two hours which included 20–25 minutes of VR sessions over 14–16 months. The study also included a preliminary introduction session and a subsequent data analysis session with participants	Once	Home-based VR intervention	A commercially available passive VR system using Oculus library environments and a bespoke active environment. Adapted Oculus Quest 2 controllers to allow for interactions such as pointing and clicking in the virtual environment through one main “trigger” button. A virtual representation of one’s hands to further facilitate interaction in the VE	Caregivers reported feeling empowered to make future design decisions and became oriented to VR use, sparking interest and ideas for future design
Harrington et al. [34]. Country: United States Design: a qualitative study with descriptive and interpretative thematic analysis	Rural person-in-context dementia simulation	10 rural female family caregivers. The interview contexts included the time they entered the simulation until respondents were interviewed within two weeks. Conversational interviewing facilitated building a rapport quickly and established authenticity. Baseline interview questions provided a uniform guide for the interviewer with variable responses based on the participants’ responses and willingness to share their stories	Discover rural family dementia caregivers’ lived experience in a virtual dementia simulation and how it affected their understanding of their family members’ daily challenges	Allow the learners to participate in a simulated AD/ADRD experience to gain the opportunity for self-discovery about their loved ones’ daily challenges by temporarily living through life and tasks through their lens	Included the time they entered the simulation until respondents were interviewed within two weeks	Ten face-to-face interviews were conducted using a conversational style that was recorded for transcription purposes. The semi-structured interviews ranged from 44–110 minutes in length and resulted in 157 pages of transcripts	Researcher-as-instrument facilitated conversational interactions and space where respondents felt safe sharing their lived experiences and meanings	Virtual dementia simulation technologies that use a person-in-context simulated experience	The person-in-context simulation provided (a) a deeper understanding of the feelings and behaviors of the PLWD and (b) a subjective meaning to the PLWD’s challenges and their complexities
Moyle et al. [35]. Country: Australia. Design: mixed-methods pilot study	Virtual reality forest (VRF)	10 PLWD, 10 family caregivers, and 9 professional caregivers. PLWD, caregivers, and staff were introduced to a VRF to increase engagement, reduce apathy, and improve mood states	To measure and describe the effectiveness of a VRF on engagement, apathy, and mood states of PLWD and explore the experiences of staff, people with dementia, and their families	To engage PLWD in an activity and thereby reduce apathy and improve mood states	Three months; sessions were 35 minutes in duration, which included a 15-minute VR experience and a 20-minute post-intervention interview. (data were also collected pre-intervention to serve as a baseline)	Once	Care facility-based VR intervention	A large interactive screen designed to immerse the user in the virtual environment. Video game technology, involving vivid graphics and motion sensors, to create an interactive and immersive environment. The imagery of the VRF was accompanied by a background soundtrack incorporating peaceful white noise and forest sounds. Microsoft Kinect® motion sensors allowed participants to interact with the scene through hand and arm movements	Overall, PLWD, family members, and aged care facility staff perceived the VRF to have a positive effect
O’Connor et al. [36]. Country: United States Design: a feasibility pilot study	Online support group for dementia caregivers in a 3D virtual environment	Seven family caregivers participated in a virtual support group with the goal of reducing stress, depression, and loneliness	To investigate a virtual online caregiver support group	To bring a caregiver support group into the home	Eight weeks (additionally, pre-and post-intervention surveys to gather psychosocial data were conducted)	Conducted weekly for one hour	Home-based VR intervention	The software program Second Life offered visual and interactive 3D features and allowed for navigation of the virtual world through simple keyboard commands	Data indicated lower levels of perceived stress, depression, and loneliness across participants. Caregivers overcame barriers to participation and had a strong sense of the group’s presence
Slater et al. [32]. Country: United Kingdom. Design: a quasi-experimental one-group pre-test post-design	Simulation-based dementia training	223 family caregivers and health professionals. The virtual dementia tour program is a replication of stage 4-5 (moderate) dementia involving sensory distortion using apparatus and cognitive confusion by requiring participants to complete simple tasks, such as folding clothes. The virtual dementia tour program uses the transformative learning techniques to place the participant in the realm of dementia and provides participants with an imagined insider’s view of the condition and an opportunity to self-reflect on the experience to help better understand what it is like living with the condition	To examine the impact of the virtual dementia tour on empathetic thinking, understanding, and person care	Use of a virtual dementia tour to improve empathetic thinking, understanding, and person care	Two hours with a 30-minute debriefing to complete	30-minute debriefing program completed the training	Not reported (NR)	Sensory distortion apparatuses through the use of a VR program and technologies	Significant positive change in empathetic understanding. Positive behavioral impact of the condition on the person. Better understanding among healthcare professionals of the isolated, fragmented, and confusing world of PLWD
Wijma et al. [37]. Country: the Netherlands. Design: a pre-/post pilot study	A VR intervention	35 family caregivers. Caregivers were asked to engage in a VR simulation movie and an e-course with the goal of increasing understanding of PLWD	To evaluate the impact of Through the D’Mentia Lens on caregivers’ understanding of PLWD	To improve the understanding of PLWD	Introduction and demo followed by a 13-minute VR simulation movie, 3 × 20-minute e-course modules, and post-testing up to three weeks later	Once	360-degree VR simulation movie and e-course that participants could follow at home	A simulation movie played on a VR device that reacted to the movements of the viewer, enabling them to look around 360 degrees	Better ability to empathize with the PLWD, increased confidence in their care task, and improved positive interaction with the PLWD

Beneficial Outcomes of Interventions

VR interventions tailored for dementia caregivers were found to have numerous benefits. Beyond imparting practical caregiving skills [32,34], these approaches serve as conduits for fostering enhanced empathy and understanding [32,34-37]. Utilizing modalities such as VR and online educational modules, caregivers acquire insights into the intricate dynamics of dementia care. Consequently, caregivers report augmented confidence and competence in their caregiving roles [32,34], concomitant with reduced levels of stress and feelings of isolation.

Empathy: Five studies (n = 5/6, 83%) showed a positive change in empathetic understanding [32,34-37]. Empathetic understanding, as demonstrated in these articles, refers to the ability to deeply comprehend and appreciate the challenges, experiences, and needs of PLWD [32,34,35,37]. This understanding often resulted from various immersive VR and AR interventions and methods. These outcomes relied on participant debriefs [32], interviews [34,37], surveys [36], and e-courses [37]. Slater et al. used sensory distortion apparatuses and cognitive confusion techniques (virtual dementia tours) on simple tasks such as folding clothes [32]. The AR helped participants understand what it feels like to live with dementia, encouraging self-reflection and an empathetic mindset [32]. Another study applied a similar method in which caregivers were enrolled in a virtual dementia simulation technology so that they could understand the challenges faced by PLWD [34]. Wijma et al. [37] used VR simulation to show a movie while Moyle et al. [35] used video game technology with graphics, motion sensors, and various sounds to immerse participants in the virtual environment [35]. Both studies showed a positive effect where PLWD experienced leisure, increased alertness, and reduced anxiety. O’Connor et al. [36] observed empathy in caregivers by creating an online virtual caregiver support group in which caregivers noted lower levels of stress, depression, and loneliness in pre and post-intervention surveys [36].

Skill-building: Skill-building in the context of the articles referred to the process of enhancing the abilities and competencies of caregivers and healthcare professionals to better understand, manage, and provide care for PLWD [32,34]. Two studies (n = 2/6, 33%) showed a better understanding of PLWD’s feelings and behaviors [32,34]. They were facilitated by sensory distortion apparatuses using VR and virtual dementia simulation technology [32,34], where each participant was questioned after the simulation.

Competence: One study (n = 1/6, 17%) showed that caregivers were more confident about their responsibilities of care and had more positive relationships with PLWD [37] after viewing a VR-simulated movie that gave them a reactive 360-degree view from the perspective of a PLWD [37]. Caregivers and PLWD were empowered to make decisions relating to VR use as they were asked about what VR design would be suitable to be used by PLWD in the future [33].

Psychological well-being: VR and AR interventions exhibited the potential to offer significant psychological well-being benefits. O’Connor et al. [36] observed a reduction in perceived stress, depression, and loneliness among caregivers. Furthermore, Wijma et al. [37] reported improved caregiver empathy, increased confidence in caregiving, and more positive interactions. Family caregivers also reported positive effects from their VR experience [35].

Barriers to Technology Acceptance

While VR and AR technologies present compelling opportunities for dementia-related interventions, there remain several barriers to its uptake and sustained adoption. Analysis revealed two barriers described below. These barriers encompass users’ technological limitations and accessibility issues.

Technological inexperience:* *Inadequate prior user experience with technology can significantly affect the usability of digital interventions [36]. This leads to increased time and effort required for participants and potential technical challenges, as illustrated by instances of users falling victim to malware from misleading links [36].

Physical capabilities of users: Another potential barrier to the use of VR and AR is the requirement for a minimum range of motion from the user, as these interventions often necessitate some level of physical movement. For instance, in their study aimed at introducing PLWD and their caregivers to VR, Flynn et al. [33] highlighted a lack of swivel chairs to support physical mobility in some home environments, which hindered user engagement with the technology.

Equipment limitations: Similarly, Moyle et al. [35] evaluating the effects of a VR noted that the required physical movement often led to fatigue in most users.

Accessibility barriers:* *The accessibility barriers faced by users, including issues with difficulties managing the download, inadequate equipment, and a lack of familiarity with specific technology, can significantly impact the effectiveness of VR interventions for family caregivers. One article (n = 1/6, 17%) described that one participant was unable to run the program due to inadequate computing equipment. Inappropriate graphics clarity in active virtual environments caused accessibility challenges for those with vision impairments [33].

Facilitators of Technology Acceptance

Several facilitators are essential for ensuring the sustained adoption of VR and AR. Notably, user-centered technology was described as ensuring that AR and VR interventions were user-friendly, engaging, and accessible, ultimately enhancing their impact on healthcare. Additionally, technological assistance, troubleshooting resources, and customization options provided necessary support for caregivers. We describe these in the sections below.

User-centered technology: One article (n=1/6, 16.6%) underscored the importance of tailoring technology to the needs and preferences of the users, ultimately contributing to the sustained success of AR and VR technologies in healthcare interventions [33]. Another study reported a substantial degree of engagement among caregivers when they were able to appropriately use the VR tool [33]. Features such as the Oculus Comfort Head Strap and glasses spacer enhance the comfort and accessibility of the technology, making it more accommodating for users, including those who wear glasses [33]. Moreover, incorporating options to use VR technology in different ways, such as the ability to use the technology in either a seated or standing position accommodates individuals with reduced standing tolerance and balance issues, making the intervention more inclusive [33]. For example, one intervention utilized a text- and graphic-based program as it could be used on most computers, ensuring accessibility even for those without advanced hardware, such as webcams and audio capabilities [36]. For studies conducted during the COVID-19 pandemic, being able to use the technology at home was described as being more supportive of caregivers who preferred to practice social distancing and avoid community events to access the intervention safely and conveniently [37].

Technological assistance: Recognizing the importance of facilitation and assessing the level of assistance required by participants ensures that the intervention is appropriately tailored to their needs [33]. Flynn et al. [33] emphasized the importance of “support the supporters” by providing caregivers with sufficient training and resources, particularly in assisting with the setup and utilization of VR. Providing instruction manuals or videos for set-up and usage, such as how to wear and remove the headset, configure controller orientation, and launch applications, was found to make it easier for users to get started [33]. Moreover, articles that described interventions that provided troubleshooting strategies for caregivers explained that these resources enabled family caregivers to address common issues with using new technology and find solutions, thus reducing potential barriers to effective use [33]. Troubleshooting resources came in various forms, including virtual support groups, which allowed caregivers to participate from their homes, overcame participation barriers and provided a sense of community and support [36].

Anonymity through avatars: One article (n = 1/16, 17%) noted that using avatars in interactions through VR and AR, rather than video-conferencing during virtual support groups that show identity, offered family caregivers anonymity [36]. The anonymity encouraged caregivers to partake in training and education without concerns about their appearance and fostered open peer-to-peer discussions about difficult experiences and emotions associated with dementia caregiving [36].

Discussion

In light of the growing challenges posed by dementia within our aging population, this scoping review sought to examine the use, effectiveness, and acceptability of VR and AR interventions in supporting family caregivers as they care for PLWD [19], with a focus on factors promoting acceptability and leading to effective outcomes. Dementia caregivers often encounter physical, mental, emotional, and social health challenges. Literature suggests that existing VR and AR interventions are effective in supporting caregivers’ well-being [38]. While traditional interventions based on in-person teaching and support have played a vital role in offering dementia caregivers the tools and skills to sustain and support them in their caregiving journey, this scoping review explores the emerging field of VR and AR innovations that may further advance caregiver education and training [37]. The current review provided promising evidence that VR and AR interventions may be associated with fostering empathy and understanding among caregivers, improving their competence, and delivering various psychological benefits, including reducing stress and depression. Furthermore, the review identifies key facilitators for sustained adoption, such as user-centered technology, technological assistance, and anonymity through avatars. Despite these positive outcomes and facilitators, the review also highlights barriers, including issues related to user technological experience and accessibility. Lastly, this review noted a lack of grey literature, possibly because VR and AR simulation training for family caregivers and their evaluation may not be widely available.

Our review noted promising outcomes of VR and AR usage on caregivers’ skill-building, empathy development, and competence enhancement, which are consistent with findings in existing literature reviews for caregivers more broadly (i.e., not exclusive to dementia caregiving, nor unpaid/family caregiving) [19]. Moreover, VR training can be conveniently administered within the home environment, underscoring its accessibility, and suggesting that it may be more readily accepted by caregivers. This convenient home-based approach aligns with the growing emphasis on providing support and resources to caregivers in a manner that is both practical and comfortable and may play a pivotal role in supporting PLWD and caregivers in aging-in-place. Our findings did differ from an existing review on the use and impact of VR simulation in dementia care education, which included articles that involved a VR intervention within a formal dementia care education setting (e.g., nursing school, caregiver training) [19]. Understanding VR and AR technology is crucial due to their convenience and flexibility, which are essential for accommodating the diverse daily routines of individuals. However, despite its potential benefits, none of the articles in our review addressed payment structures that could alleviate these costs. This absence underscores a critical avenue for further exploration within this field. Evaluations of initiatives such as inclusion in assistive device programs or piloting schemes within healthcare organizations could subsidize expenses for users, potentially enhancing accessibility [39]. The lack of attention to subsidizing these costs in the current literature accentuates equity concerns, aligning with discussions in recent articles on equity in AR/VR utilization [40]. Addressing the financial aspect is pivotal for widespread adoption and meaningful utilization, necessitating further exploration and policy considerations to ensure equitable access to these transformative technologies targeted at PLWD and their caregivers.

To ensure the acceptability of the use of VR in caregiver training, barriers identified in the review should be adequately addressed. One of the significant barriers to the adoption of VR in caregiver training is users’ inadequate prior technological experience, which can lead to challenges with setting up and using hardware and software. In particular, caregiver participants who had limited experience with technology encountered increased time and effort requirements when using VR technology [35]. Although not an objective of our review, it is also worth highlighting that none of the studies investigated the creation of culturally competent VR and AR programming. This represents a critical gap in exploring these innovations. Understanding the viewpoints of ethnocultural and other minority groups is crucial to providing appropriate content, underscoring the need for co-design approaches that consider these factors. One example of how this has been implemented is a VR-assisted psychotherapy within the Inuit population that integrated culturally sensitive techniques to ensure relevance and effectiveness within their specific cultural context [41]. By engaging caregivers in the co-design and testing of VR and AR interventions, their needs, preferences, and limitations can be incorporated into the technology [42]. Co-design methodology, in particular, allows for continuous usability testing, in which caregivers can provide feedback on their experiences with the technology [42]. This feedback loop or iterative development process may help identify and address issues related to user engagement, physical requirements, and fatigue. Moreover, a co-design approach can enable caregivers to report their security concerns or issues, allowing for quick responses and improvements in the safety of VR and AR applications. As VR and AR also offer different user experiences and levels of immersion, understanding these distinctions from the caregiver perspective may be vital for ensuring that the training programs are engaging and relevant to the needs of caregivers. Comparative studies are encouraged to provide insights into which technology is more suitable for specific training objectives and user groups.

The absence of a grounding in pedagogical principles or behavioral change theory in the reviewed articles is a noteworthy limitation of the current state of existing VR and AR technologies. These frameworks are often considered important for designing effective educational and behavior-change programs [43]. In addressing the adoption of technology for caregiving roles, it is crucial to not only target behavior change but also address attitudes, especially among family caregivers of PLWD who might be hesitant toward technology [44]. Existing literature has found that individuals willing to use VR are significantly younger, and, in turn, underscores the importance of tailoring interventions to bridge this digital divide among older individuals, emphasizing the need for targeted approaches to enhance their self-efficacy and confidence in utilizing innovative technologies for caregiving purposes [45]. Strategies aimed at engaging these caregivers, particularly those averse to utilizing innovations, become pivotal. Without a clear understanding of pedagogical principles and behavioral change theory, it becomes challenging to ensure that the training methods and content align with best practices for adult learning and behavioral change, which, in turn, potentially limits the impact of the interventions on caregiver knowledge, attitudes, and caregiving practices. VR and AR technology is poised to serve as adjunctive support, complementing and enhancing existing best-use cases within the realm of dementia care and support [46]. Pedagogical principles and behavioral change theories can inform the customization and personalization of training programs. As carers represent a heterogeneous population and have diverse learning needs and abilities [47], understanding these principles can help tailor the training to meet individual requirements. Moreover, behavioral change theories offer strategies for sustaining new behaviors and habits [48]. Thus, to support effective, evidence-based interventions, future studies should consider involving caregivers in co-designing VR and AR technologies utilizing the pedagogical principles and behavioral change theories and comparing their efficacy to existing interventions to determine the role of such theories in caregiver interventions. Behavioral change theory can guide the evaluation of long-term behavior change in the context of dementia caregiving [49]. Future studies should explore the efficacy of such tailored interventions, comparing them to existing methods and assessing the role of pedagogical principles and behavioral change theories in influencing caregiver interventions.

Limitations

The review was limited to articles published in the English language and those available from January 2010 to October 2023. This restriction might have excluded potentially relevant research published in languages other than English or before January 2010. Consequently, valuable insights from non-English literature or older publications might have been omitted. While efforts were made to identify grey literature sources, it is important to acknowledge that grey literature on clinical interventions, such as VR and AR for dementia caregiving, can be less accessible through traditional search methods. Although the review did not discover any grey literature, it is essential to recognize that grey literature may exist, and it might include important unpublished or non-standard format studies that were not included in this review. Lastly, although the review made use of multiple databases, there is always the possibility that some relevant sources may not have been included in the search strategy. This limitation is mitigated by the hand-search of the reference lists of included articles and forward searching, yet some potentially pertinent studies may still have been overlooked. Lastly, the failure to distinguish between AR and VR in the reviewed articles is a methodological shortcoming that can lead to confusion in the analysis and makes it difficult to assess which technology is more effective for specific training objectives.

## Conclusions

This scoping review of the literature on the use of VR and AR interventions for family caregivers of PLWD offers valuable insights into the potential benefits and challenges of employing these technologies in the context of dementia care. The review encompassed a range of research designs and approaches from various countries, providing a comprehensive overview of the current state of research in this field. The findings suggest that VR and AR interventions have the potential to positively impact family caregivers in several ways: enhance empathy, understanding, and skill-building among caregivers, ultimately leading to improved care for PLWD. Notably, family caregivers who participated in these interventions reported increased confidence, competence, and resilience in their caregiving roles. Furthermore, the interventions demonstrated a capacity to reduce stress, depression, and loneliness among caregivers, which is crucial for maintaining their well-being. However, several barriers and challenges to acceptability were identified, including issues related to user experience, technological limitations, and accessibility. To ensure the sustained acceptability of VR and AR interventions, user-centered technology, technological assistance, and the customization of care delivery are essential.

## References

[1] Ismail Z, Black SE, Camicioli R (2020). Recommendations of the 5th Canadian Consensus Conference on the diagnosis and treatment of dementia. Alzheimers Dement.

[2] Birckhead B, Khalil C, Liu X (2019). Recommendations for methodology of virtual reality clinical trials in health care by an international working group: iterative study. JMIR Ment Health.

[3] Brodaty H, Donkin M (2009). Family caregivers of people with dementia. Dialogues Clin Neurosci.

[4] Pleasant M, Molinari V, Dobbs D, Meng H, Hyer K (2020). Effectiveness of online dementia caregivers training programs: a systematic review. Geriatr Nurs.

[5] Sadavoy J, Sajedinejad S, Duxbury L, Chiu M (2022). The impact on employees of providing informal caregiving for someone with dementia. Aging Ment Health.

[6] Pinquart M, Sörensen S (2006). Helping caregivers of persons with dementia: which interventions work and how large are their effects?. Int Psychogeriatr.

[7] Vandepitte S, Putman K, Van Den Noortgate N, Verhaeghe S, Annemans L (2019). Effectiveness of an in-home respite care program to support informal dementia caregivers: a comparative study. Int J Geriatr Psychiatry.

[8] Kokorelias KM, Gignac MA, Naglie G, Rittenberg N, MacKenzie J, D'Souza S, Cameron JI (2022). A grounded theory study to identify caregiving phases and support needs across the Alzheimer's disease trajectory. Disabil Rehabil.

[9] Carter G, Monaghan C, Santin O (2020). What is known from the existing literature about peer support interventions for carers of individuals living with dementia: a scoping review. Health Soc Care Community.

[10] Liu J, Lou Y, Cheung ES, Wu B (2022). Use of tangible, educational and psychological support services among Chinese American dementia caregivers. Dementia (London).

[11] Hicks B, Konovalova I, Myers K, Falconer L, Board M (2023). Taking ‘a walk through dementia’: exploring care home practitioners’ experiences of using a virtual reality tool to support dementia awareness. Ageing Soc.

[12] Stargatt J, Doube W, Bhar S, Petrovich T, McGuire L, Willison A (2022). Increasing understanding of environmental modifications using the Virtual Dementia Experience for professional carers of people living with dementia. Gerontol Geriatr Educ.

[13] Stargatt J, Bhar S, Petrovich T, Bhowmik J, Sykes D, Burns K (2021). The effects of virtual reality-based education on empathy and understanding of the physical environment for dementia care workers in Australia: a controlled study. J Alzheimers Dis.

[14] Brown EL, Agronin ME, Stein JR (2020). Interventions to enhance empathy and person-centered care for individuals with dementia: a systematic review. Res Gerontol Nurs.

[15] Yang YH, Situmeang RF, Ong PA, Liscic RM (2022). Application of virtual reality for dementia management. BSA.

[16] Carroll J, Hopper L, Farrelly M, Lombard-Vance R, Bamidis PD, Konstantinidis EI (2021). A scoping review of augmented/virtual reality health and wellbeing interventions for older adults: redefining immersive virtual reality. Front Virtual Real.

[17] Jones C, Jones D, Moro C (2021). Use of virtual and augmented reality-based interventions in health education to improve dementia knowledge and attitudes: an integrative review. BMJ Open.

[18] Dickinson R, Kimball J, Fahed M, Chang T, Sekhon H, Vahia IV (2023). Augmented reality (AR) in dementia care: understanding its scope and defining its potential. Am J Geriatr Psychiatry.

[19] Hirt J, Beer T (2020). Use and impact of virtual reality simulation in dementia care education: a scoping review. Nurse Educ Today.

[20] García-Betances RI, Arredondo Waldmeyer MT, Fico G, Cabrera-Umpiérrez MF (2015). A succinct overview of virtual reality technology use in Alzheimer's disease. Front Aging Neurosci.

[21] Curran GM, Bauer M, Mittman B, Pyne JM, Stetler C (2012). Effectiveness-implementation hybrid designs: combining elements of clinical effectiveness and implementation research to enhance public health impact. Med Care.

[22] Damschroder LJ, Reardon CM, Widerquist MA, Lowery J (2022). The updated Consolidated Framework for Implementation Research based on user feedback. Implement Sci.

[23] Arksey H, O'Malley L (2005). Scoping studies: towards a methodological framework. Int J Soc Res Methodol.

[24] Levac D, Colquhoun H, O'Brien KK (2010). Scoping studies: advancing the methodology. Implement Sci.

[25] Tricco AC, Lillie E, Zarin W (2018). PRISMA Extension for Scoping Reviews (PRISMA-ScR): checklist and explanation. Ann Intern Med.

[26] Bramer WM, Giustini D, de Jonge GB, Holland L, Bekhuis T (2016). De-duplication of database search results for systematic reviews in EndNote. J Med Libr Assoc.

[27] Babineau J (2014). Product review: Covidence (systematic review software). JCHLA/JABSC.

[28] Haddaway NR, Grainger MJ, Gray CT (2022). Citationchaser: a tool for transparent and efficient forward and backward citation chasing in systematic searching. Res Synth Methods.

[29] Park CU, Kim HJ (2015). Measurement of inter-rater reliability in systematic review. Hanyang Med Rev.

[30] McHugh ML (2012). Interrater reliability: the kappa statistic. Biochem Med.

[31] Assarroudi A, Heshmati Nabavi F, Armat MR, Ebadi A, Vaismoradi M (2018). Directed qualitative content analysis: the description and elaboration of its underpinning methods and data analysis process. J Res Nurs.

[32] Slater P, Hasson F, Moore K, Sharkey F (2021). Simulated based dementia training: impact on empathic understanding and behaviour among professionals and carers. Clin Simul Nurs.

[33] Flynn A, Barry M, Qi Koh W, Reilly G, Brennan A, Redfern S, Casey D (2022). Introducing and familiarising older adults living with dementia and their caregivers to virtual reality. Int J Environ Res Public Health.

[34] Harrington CC, Hardin SR, Cacchione PZ, Roberson DW, Neil JA (2022). Rural family caregivers’ discoveries following a person-in-context dementia simulation. Online J Rural Nurs Health Care.

[35] Moyle W, Jones C, Dwan T, Petrovich T (2018). Effectiveness of a virtual reality forest on people with dementia: a mixed methods pilot study. Gerontologist.

[36] O'Connor MF, Arizmendi BJ, Kaszniak AW (2014). Virtually supportive: a feasibility pilot study of an online support group for dementia caregivers in a 3D virtual environment. J Aging Stud.

[37] Wijma EM, Veerbeek MA, Prins M, Pot AM, Willemse BM (2018). A virtual reality intervention to improve the understanding and empathy for people with dementia in informal caregivers: results of a pilot study. Aging Ment Health.

[38] Cheng ST (2017). Dementia caregiver burden: a research update and critical analysis. Curr Psychiatry Rep.

[39] Brooke V, McDonough J, Hardy S (2006). Benefit specialists: key resources for supporting the use of Social Security work incentives to fund assistive technology. J Vocat Rehabil.

[40] Farrell K, MacDougall D (2023). An Overview of Clinical Applications of Virtual and Augmented Reality: Emerging Health Technologies. https://canjhealthtechnol.ca/index.php/cjht/article/view/EH0111/1264..

[41] Seon Q, Mady N, Yang M (2023). A virtual reality-assisted cognitive behavioral therapy for and with Inuit in Québec: protocol for a proof-of-concept randomized controlled trial. JMIR Res Protoc.

[42] Latulippe K, Hamel C, Giroux D (2020). Co-design to support the development of inclusive eHealth tools for caregivers of functionally dependent older persons: social justice design. J Med Internet Res.

[43] Klodnicka KK, Ducharme FC, Giroux F (2011). A psycho-educational intervention focused on communication for caregivers of a family member in the early stage of Alzheimer’s disease: results of an experimental study. Dementia.

[44] Guisado-Fernández E, Giunti G, Mackey LM, Blake C, Caulfield BM (2019). Factors influencing the adoption of smart health technologies for people with dementia and their informal caregivers: scoping review and design framework. JMIR Aging.

[45] Mosadeghi S, Reid MW, Martinez B, Rosen BT, Spiegel BM (2016). Feasibility of an immersive virtual reality intervention for hospitalized patients: an observational cohort study. JMIR Ment Health.

[46] Cerritelli F, Chiera M, Abbro M, Megale V, Esteves J, Gallace A, Manzotti A (2021). The challenges and perspectives of the integration between virtual and augmented reality and manual therapies. Front Neurol.

[47] Mastel-Smith B, Stanley-Hermanns M (2012). "It's like we're grasping at anything": caregivers' education needs and preferred learning methods. Qual Health Res.

[48] Kwasnicka D, Dombrowski SU, White M, Sniehotta F (2016). Theoretical explanations for maintenance of behaviour change: a systematic review of behaviour theories. Health Psychol Rev.

[49] Heimlich JE, Ardoin NM (2008). Understanding behavior to understand behavior change: a literature review. Environ Educ Res.

