# Correction: Use of Virtual Reality and Augmented Reality Technologies to Support Resilience and Skill-Building in Caregivers of Persons With Dementia: A Scoping Review

**DOI:** 10.7759/cureus.c260

**Published:** 2025-08-26

**Authors:** Kristina M Kokorelias, Mary Chiu, Sayani Paul, Lynn Zhu, Nusrat Choudhury, Cole G Craven, Adam Dubrowski, Tyler Redublo, Bill Kapralos, Michael S.D. Smith, Adriana Shnall, Joel Sadavoy, Amer Burhan

**Affiliations:** 1 Section of Geriatrics, Sinai Health and University Health Network, Toronto, CAN; 2 Rehabilitation Sciences Institute, Temerty Faculty of Medicine, University of Toronto, Toronto, CAN; 3 Research & Academics, Ontario Shores Centre for Mental Health Sciences, Whitby, CAN; 4 Faculty of Health Sciences, Ontario Tech University, Oshawa, CAN; 5 Medical Devices, National Research Council Canada, Boucherville, CAN; 6 Computer Science, Ontario Tech University, Oshawa, CAN; 7 maxSIMhealth Group, Ontario Tech University, Oshawa, CAN; 8 Translational Research Program, Temerty Faculty of Medicine, University of Toronto, Toronto, CAN; 9 Software Informatics Research Centre, Ontario Tech University, Oshawa, CAN; 10 Medical Devices, National Research Council Canada, Winnipeg, CAN; 11 The Koschitzky Centre for Innovations in Caregiving, Baycrest Centre, Toronto, CAN; 12 Factor Inwentash Faculty of Social Work, University of Toronto, Toronto, CAN; 13 Department of Geriatric Psychiatry, Mount Sinai Hospital, Toronto, CAN; 14 Department of Psychiatry, Temerty Faculty of Medicine, Unviersity of Toronto, Toronto, CAN; 15 Applied Mental Health, Ontario Shores Centre for Mental Health Sciences, Toronto, CAN; 16 Department of Psychiatry, Temerty Faculty of Medicine, University of Toronto, Toronto, CAN

This article has been corrected to fix the following typos in the PRISMA diagram and a statement in the second paragraph of the 'Beneficial Outcomes of Interventions' section:

- In the PRISMA diagram, "Reports excluded" has been corrected from 104 to 106.
- In the first sentence of the second paragraph of the 'Beneficial Outcomes of Interventions' section, “Empathy: Six studies (n = 6/10, 60%) showed...” has been corrected to “Empathy: Five studies (n = 5/6, 83%) showed…”

